# Comparison of Liquid Chromatography- and Nano-Electrospray Ionization-Mass Spectrometry Approaches for Single-Cell Metabolomics

**DOI:** 10.1021/acs.analchem.5c06318

**Published:** 2026-03-18

**Authors:** Abigail Cook, Claire Davison, Jordan Pascoe, Harpreet Atwal, George Mayson, Ahmed Ali, Dany JV Beste, Melanie Bailey

**Affiliations:** † Faculty of Engineering and Physical Sciences, 3660 University of Surrey, Guildford GU2 7XH, U.K.; ‡ Department of Infectious Diseases,King’s College London Guy’s Hospital, London SE1 9RT, U.K.; § Faculty of Health and Medical Sciences, 3660 University of Surrey, Guildford GU2 7XH, U.K.; ∥ Metabolomics & Analytics Centre LACDR, 195335 Leiden University, 2300 RA Leiden, The Netherlands

## Abstract

Live single-cell metabolomics is a rapidly growing area of research, which offers the potential to provide unique insights into cellular function and heterogeneity. Single-cell isolation approaches based on capillary sampling are in principle compatible with either nano-electrospray ionization-mass spectrometry (nano-ESI-MS), where the cell is lysed and sprayed directly into a mass spectrometer, or liquid chromatography–mass spectrometry (LC-MS) for metabolomics analysis. However, there are no data indicating which approach can provide the best performance (metabolite coverage, reproducibility and sensitivity) for single-cell metabolomics. In this work, we have developed and then compared two semitargeted metabolomics methods (direct nano-ESI-MS and LC-MS) for detecting amino acids and other hydrophilic metabolites in single macrophages. Interestingly, our results show that, even when using analytical-flow LC-MS, the coverage of metabolites is superior to the nano-ESI-MS method. We applied both methodologies to single THP-1 macrophages infected with fluorescent *Mycobacterium bovis* bacillus Calmette-Guérin (BCG), the vaccine strain of *Mycobacterium tuberculosis*. Infected cells were identified under a microscope and sampled into glass capillaries. Our results show that the LC-MS approach provides a much clearer distinction between infected and control cells than using nano-ESI-MS. LC-MS detected enrichment of several compounds in infected cells, including methionine, cysteine and taurine, highlighting reprogramming of sulfur metabolism during mycobacterial infection. These findings establish a robust analytical framework for spatially resolved single-cell metabolomics and underscore its potential for uncovering infection-driven metabolic heterogeneity, with broad applications in infectious disease research, drug discovery, and clinical diagnostics.

## Graphical abstract

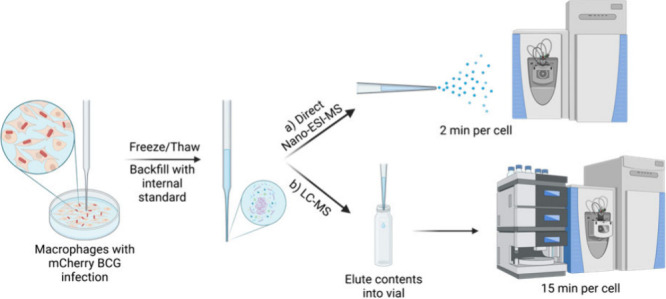

Metabolomics and single-cell isolation represent two cutting-edge approaches in modern biology, and their integration unlocks unprecedented insights into cellular heterogeneity and processes. Through profiling of small molecules, metabolomics provides a direct snapshot of biochemical activity that reflects real-time cellular processes. Specifically, it reveals the functional outcomes of gene expression, enzyme activity, and environmental interactions.^1,2^ Traditional bulk analyses average signals across thousands to millions of cells, masking variation between individuals.^3^ In contrast, single-cell analysis is essential for resolving the variability hidden in bulk populations, enabling the study of different cell types and their dynamic responses to environmental or therapeutic stimuli.^4^ Single-cell metabolomics offers the potential to reveal how distinct cells, even from the same cell line, exhibit cell-to-cell biochemical heterogeneity, enabling the dissection of metabolic variation in disease, to pave the way for precision diagnostics and personalized therapies.^5–7^

Both the performance and interpretability of single-cell metabolomics are shaped by the sampling strategy. Recent advances have seen several single-cell isolation methods evolve, each with their own application-specific advantages and limitations.^8^ Mass spectrometry imaging (MSI) has emerged as a powerful entry point into single-cell metabolomics, offering label-free, spatially resolved molecular data. MSI techniques such as matrix-assisted laser desorption ionization (MALDI), desorption electrospray ionization (DESI) and secondary ion mass spectrometry (SIMS) can now attain single-cell resolution, enabling spatial mapping of cellular populations of lipids and metabolites.^9–11^ These techniques have the disadvantage that cells cannot be sampled live and lack chemical depth and quantitation offered by chromatographic methods.^5^

By contrast, flow cytometry or droplet microfluidics enable high-throughput sorting of individual eukaryotic or prokaryotic cells, which can then be lysed and directed into electrospray ionization (ESI) compatible platforms such as liquid chromatography (LC)-MS or capillary electrophoresis (CE)-MS. These sampling methods excel in scalability and statistical power but lack any spatial resolution, meaning that information on cellular interactions and microenvironments is not captured.^12,13^

Live-cell capillary-based sampling uses micro or nano glass capillaries to aspirate individual cells or subcellular compartments directly from living systems.^14^ This enables spatially resolved live-cell sampling, which facilitates further biological understanding of cells in their native state. This technique is ideally suited for direct nano-electrospray ionization-mass spectrometry (nano-ESI-MS) as the sampling capillary can be utilized as a nano-ESI emitter to spray the sample directly into the mass spectrometer.^9,15^ While most capillary-based cell sampling has utilized home-built micromanipulators or manual aspiration,^16–19^ an automated system was commercialized in 2022, which will improve standardization across laboratories and reduce the human errors involved.^5,20^

Nano-ESI-MS enables rapid, low-volume analysis with minimal sample preparation, making it attractive for high-throughput and spatial metabolomics. However, a limitation of nano-ESI-MS is poor reproducibility, ion suppression and limited openly available data analysis software.^21,22^ We have previously demonstrated that lipids can be detected and annotated in single cells using LC-MS;^20,23–25^ this offers chromatographic separation to reduce ion suppression and enables accurate quantification, as well as untargeted compound annotation using many open-access data analysis solutions.^26–28^ However, these workflows suffer from transfer losses from the capillary tip to the LC-MS vial, sample dilution, and low throughput due to chromatographic separation steps.^24^ It is therefore timely to compare the performance of LC-MS and nano-ESI-MS methods for single-cell analysis.

This work describes the development and optimization of nano-ESI-MS and LC-MS methods for the analysis of hydrophilic metabolites in live single cells. The two analysis methods are compared in terms of their sensitivity, precision, linearity to targeted amino acids, and their coverage of metabolite features in cell extracts. The methodologies are then applied to determine single-cell metabolic signatures of a tuberculosis macrophage model of infection.

Tuberculosis (TB) is the leading cause of human death from a single infectious agent. The causative agent of TB, *Mycobacterium tuberculosis* (Mtb), spends much of its life cycle living within macrophages, therefore understanding the interactions between this pathogen and its human host cell is a key focus of TB research.^29,30^ Recent studies have revealed metabolic crosstalk between host and pathogen play a key role in the disease process.^31,32^ Indeed, bulk metabolomics measurements have enabled new insights into the host-pathogen interaction, however, these only provide population averages when cells are highly heterogeneous.^33–35^ By combining these host-pathogen models with single-cell approaches, deeper insights into tuberculosis pathogenesis and host immunity can be uncovered.

Here we apply the developed nano-ESI- and LC-MS methods to infected and unexposed control cells, before comparing their ability to detect metabolic perturbations that arise as a consequence of infection. To our knowledge, this is the first comparison of nano-ESI-MS and LC-MS for single-cell metabolomics and provides a practical framework for the selection of analysis method.

## Experimental Section

### Chemicals and Reagents

THP-1 and *Mycobacterium bovis* bacillus Calmette-Guérin (BCG) cells were obtained from ATCC, USA. Roswell Park Memorial Institute (RPMI) 1640 media and fetal bovine serum (FBS) were acquired from Merck, UK and Thermo Fisher Scientific, UK respectively. Middlebrook 7H9 medium was purchased from Sigma-Aldrich, UK and supplemented with glycerol (Thermo Scientific, UK), Tween80 (Sigma-Aldrich, UK) and Albumin Dextrose Catalase (ADC) (Remel, USA). Dulbecco’s Phosphate Buffered Saline (DPBS) with calcium (Ca^2+^) and magnesium (Mg^2+^) was purchased from Gibco, USA.

Solvents including acetonitrile (ACN), water (H_2_O), methanol (MeOH) and formic acid (FA) were Optima LC-MS grade from Fisher Scientific, UK. For method development, Honeywell, UK and Romil, UK brands were also analyzed. Both mixed nonlabeled amino acid (aa) standard and mixed deuterated amino acid (D-aa) standard were purchased from Merck, UK.

### THP-1 Macrophage Differentiation and Infection

To study the Mtb host-pathogen interaction safely, THP-1 monocytes were derived into macrophage-like cells and infected with attenuated *M. bovis* BCG. These accessible category 2 models are ideal for simulating infection while minimizing biosafety risks.^36–40^ For this work, we used an mCherry strain of BCG containing an episomal plasmid encoding the monomeric red fluorescent protein mCherry, under the control of the *smyc* constitutive promoter, so that the bacteria could be easily visualized by fluorescence microscopy.^41,42^

An estimated 1 × 10^6^ THP-1 cells were seeded into two 35 mm glass bottom μ-dishes (ibidi GmbH, Germany) along with complete RPMI, supplemented with 50 ng/mL phorbol 12-myristate 13-acetate (PMA). The dishes were incubated at 37 °C and 5% CO_2_ for 72 h for PMA-induced macrophage differentiation to take place. Meanwhile, mCherry BCG cultured in Middlebrook 7H9 supplemented with 0.5% glycerol, 0.2% (v/v) TWEEN 80, and 5% (v/v) Albumin Dextrose Catalase (ADC), incubated at 37 °C and 90 rpm. One dish of THP-1 macrophages was dosed with mCherry BCG at a multiplicity of infection (MOI) of 10:1 and incubated for 4 h. This was the infected dish. The extracellular bacteria were removed by washing with media and DPBS before adding 2 mL DPBS with Ca^2+^ and Mg^2+^ prior to single-cell isolation. The second dish, used as a control, underwent the same process without addition of BCG, named control unexposed dish.

### Single-Cell Imaging and Isolation

Capillary single-cell sampling was completed using the Single Cellome SS2000 (Yokogawa Corporation, Japan). The incubator was set to 37.5 °C, with 5% CO_2_ and humidity on. The cells were imaged under confocal microscopy pre and post sampling, utilizing fluorescence imaging to locate mCherry BCG (Ex 561 nm, Em 617/73 nm).

Capillary tips with an internal diameter of 10 μm (Yokogawa, Japan) with filament (for nano-ESI-MS) and without filament (for LC-MS) were used to aspirate single THP-1 macrophages using presampling, sampling and postsampling pressures of 1.00, −15.90 and 0.00 kPa, respectively. 40 single macrophages containing mCherry BCG were sampled from the infected dish. 40 control macrophages were also collected from the unexposed dish. Each tip was placed on dry ice immediately after sampling. Once sampling was complete, the capillaries were transferred to a −80 °C freezer until mass spectrometry analysis. Blank samples were collected by sampling from a dish containing only DPBS with Ca^2+^ and Mg^2+^ at random positions and stored as described for single cells.

### Sample Preparation

#### THP-1 Metabolite Extract

A cell metabolite extract was generated using the following protocol to enable method optimization. PMA induced THP-1 derived macrophages were deadhered using a cell scraper and subsequently centrifuged at 300 × *g* for 5 min. The supernatant was discarded, then cells were resuspended in RPMI for cell counting. Once counted, the cells were centrifuged again, and the pellet was resuspended in 1 mL of 2:2:1 MeOH:ACN:H_2_O with D-aa internal standard (Supporting Information Table S1) and transferred to a 2 mL autosampler vial. The resuspension underwent three freeze-thaw cycles in liquid nitrogen, then centrifuged at 20,000 × *g* for 5 min in an Eppendorf tube. The supernatant was passed through a 0.22 μm Spin-X centrifuge filtration tube (Corning Incorporated, USA) at 15,000 × *g*. The eluent was transferred to a 2 mL autosampler vial and frozen at −80 °C for future use.

### Standards and Single Cells

For method development, nonlabeled aa standard was diluted to 1:5000 (Supporting Information Tables S2 and S3) in 1:1 H_2_O:ACN + 0.1% FA. THP-1 metabolite extracts were diluted to the 50-, 10-, and 1-cell levels, in 1:1 H_2_O:ACN + 0.1% FA. Blanks were 1:1 H_2_O:ACN + 0.1% FA. All samples were mixed in equal parts with 1:25 mixed D-aa internal standard in 1:1 H_2_O:ACN + 0.1% FA, with a second blank left pure to assess the background throughout each analysis. All standards, cell extracts and blanks were added to 0.3 mL QSert vials (Supelco, UK) for LC-MS and to 10 μm nanospray emitters with filament for nano-ESI-MS.

An equally spaced 9-point calibration line (2.8–500 pM) of aa standard was prepared with the addition of D-aa internal standard, which was diluted 1:50. The same calibration standard stocks were used in both LC-MS and nano-ESI-MS analyses.

Single cells and blank DPBS in 10 μm capillary tips, with filament, were thawed for nano-ESI-MS analysis. The capillaries were then backfilled with 5 μL 1:50 D-aa internal standard in 1:1 H_2_O:ACN + 0.1% FA using GELoader pipet tips (Calibre Scientific, UK). Each tip was centrifuged at 1000 × *g* for 1 min to remove air bubbles prior to analysis using 3D printed capillary holders (University of Leiden, Netherlands).

For LC-MS, single cells and blank DPBS were prepared in the same way as for nano-ESI-MS, with the exception of the internal standard, which was 1:16.7 mixed D-aa. The contents of the tips were then eluted into QSert autosampler vials using a 10 mL gas syringe (Hamilton, UK) and syringe driver (KD Scientific Incorporated, USA)^24^ before the addition of 10 μL 1:1 H_2_O:ACN + 0.1% FA, diluting the D-aa internal standard to 1:50.

### Nano-ESI-MS

A nanospray ionization source (IonMax, Thermo Fisher Scientific, USA) was fitted onto the front of a Q-Exactive Plus Orbitrap (Thermo Fisher Scientific, USA). Cameras were fitted into the source to ensure each tip was inserted correctly, repeatably and the end of each tip was 2 mm away from the source inlet. Each capillary tip was manually loaded into the source and the *xyz* coordinates of the tip were set to 15, 17, and 32 mm, respectively. Each acquisition was operated in positive mode with a scan range of 50–750 *m*/*z* at a resolution setting of 140,000 at 200 *m*/*z*. The AGC target was 1e^6^ ions and maximum injection time of 256 ms. The spray voltage was 2 kV, and the capillary temperature was set to 275 °C. Finally, the S-lens radiofrequency value was set to 50 au. The contents of the nanospray emitter were sprayed into the mass spectrometer for 2 min, with a 0.1 min voltage delay.

### LC-MS

Ultrahigh performance liquid chromatography was carried out using an UltiMate 3000 (Thermo Fisher Scientific, USA) instrument. Chromatographic separation was achieved using a BEH amide (150 × 2.1 mm, 1.7 μm) column (Waters, USA). The HILIC separation method was adapted from previous work in bulk metabolomics.^43^ The mobile phases were A: H_2_O with 0.1% FA, and B: ACN with 0.1% FA. A simple gradient elution over 15 min started with 99% B for 0.5 min which then linearly decreased to 30% over 7 min before restoring 99% at 7.6 min allowing the column to re-equilibrate for 7.4 min. The flow rate was 0.4 mL/min.

The Q-Exactive Plus Orbitrap was equipped with a heated electrospray ionization (HESI) source (Thermo Fisher Scientific, USA) and was operated in positive mode. A scan range of 50–750 *m*/*z* at a resolution setting of 140,000 at 200 *m*/*z* was used. The AGC target was 1e^6^ ions and maximum injection time of 256 ms. The spray voltage was 4 kV, and the capillary temperature was set to 275 °C. The sheath, auxiliary and sweep gas flow rates were 60, 8, and 0 au, respectively. The S-lens radiofrequency value was set to 50 au and auxiliary gas heater to 250 °C.

### Data Analysis

For targeted analysis of amino acids, nano-ESI-MS and LC-MS data were preprocessed through TraceFinder (Thermo Fisher Scientific, USA). Amino acid peak areas and intensities for LC- and nano-ESI-MS respectively were blank corrected using PBS with Ca^2+^/Mg^2+^ and normalized to the corresponding internal standard analyte.

For untargeted analysis, both data sets were analyzed through MS-DIAL 5.3 (RIKEN, Japan). The features were annotated using a reference library of endogenous metabolites from the Human Metabolome Database (HMDB, https://hmdb.ca/) and MS-FINDER 3.7 (RIKEN, Japan) was used for formula prediction.

Both data sets were imported to RStudio 2025.05.1 + 513 (R 4.3, Posit, USA), Prism10 (GraphPad Software Incorporated, USA) and MetaboAnalyst 6.0 (https://www.metaboanalyst.ca/home.xhtml) for statistical analysis and figure production. The data sets were log10 transformed and auto scaled for analysis in MetaboAnalyst. For statistical testing, either a nonparametric Wilcoxon matched-pairs signed rank *t* test or multiple nonparametric Mann-Whitney U tests, with Holm-Šídák correction for multiple comparisons, were used to assess differences between the analytical replicates.^44–47^

## Results

### Development of an Analytical Flow LC-MS Method for Targeted and Untargeted Single-Cell Metabolomics

We combined two bulk metabolomics application notes from Waters^43^ and Thermo Fisher Scientific^48^ as a starting method for single-cell HILIC-LC-MS (Base Method). First, the sensitivity of the method to low concentration amino acids was optimized by adjusting the HESI source parameters (spray voltage, capillary temperature, sheath, auxiliary and sweep gas flow rates, S-lens radiofrequency and auxiliary gas) using 0.5 nM aa standard (Optimized Method). The Base and Optimized methods were assessed using THP-1 metabolite extracts diluted to 50-, 10- and 1-cell concentrations analyzed in triplicate. The Optimized Method provided better signal-to-noise ratio (S/N), reduced variability of amino acid standards, and a greater number of untargeted features detected in the cell extract when compared to Base Method (Supplementary Figures S1 and S2). The Optimized Method was therefore applied for subsequent analyses.

Detecting such low concentration analytes (pM-nM) within single cells requires all background interferences to be minimized, and so the effect of three different solvent brands was assessed. We compared the S/N of amino acids in 0.5 nM aa standard in triplicate (Supplementary Figure S3). Brand 3 solvents gave superior sensitivity compared to Brands 1 and 2 and so was used in subsequent analyses.

To transfer the sampled cell from the capillary tip to the LC-MS system, the contents must first be eluted onto the inner wall of the LC-MS vial. This step introduces potential analyte loss due to adsorption to the vial surface and residual retention within the capillary. To evaluate and minimize this loss, two elution strategies were compared: (1) a “Wash” method, in which 5 μL of a 1:50 D-aa standard was eluted into an empty vial followed by a 10 μL mobile phase rinse; and (2) a “No Wash” method, where 5 μL D-aa standard was eluted directly into a vial preloaded with 10 μL mobile phase. In both cases, recovery was assessed relative to the reference standard introduced directly into the vial by pipet. The Wash method yielded a higher average recovery (93%) compared to the No Wash method (85%) and was therefore adopted for all subsequent experiments (see Supplementary Figure S4).

Finally, we aimed to exploit the high mass resolution of the Orbitrap to detect a greater number of metabolite features. However, because the Orbitrap analysis time increases with the mass resolution, there is a trade-off between resolution and gaining sufficient scans across a chromatographic peak. The peak areas of amino acids in single-cell-level THP-1 extract analyzed at 70,000 and 140,000 resolutions at 200 *m*/*z* are compared in Supplementary Figure S5. A Mann-Witney U *t* test revealed no significant difference in the normalized peak areas between the two methods. However, there was a significant increase in the number of features detected when using a resolution of 140,000 compared to 70,000 (Supplementary Figures S6 and S7), therefore 140,000 was used for future analyses.

### Development of a Nano-ESI-MS Method for Targeted and Untargeted Metabolomics

We used the mass spectrometer parameters optimized for LC-MS analysis as a starting point for nano-ESI-MS method development. The nanospray source parameters (*x,y,z* position, spray voltage, S-lens radiofrequency, and capillary temperature) were optimized by analysis of a 0.5 nM aa standard and assessing the total ion chromatogram (TIC) and amino acid intensities. Supplementary Figure S8 displays the change in TIC intensity when the position of the emitter is moved in *x,y,z* directions.

Three resolutions (70,000, 140,000 and 280,000 at 200 *m*/*z*) were assessed for their ability to detect both amino acids and untargeted metabolites using a single-cell level THP-1 metabolite extract. Supplementary Figures S9 and S10 show that, while the mass resolution setting had no impact on the peak intensities of each amino acid, 140,000 produced the greatest number of total feature hits, with no significance on the average number of features (Supplementary Figure S11). Resolution was therefore set to 140,000 for subsequent comparison of nano-ESI- and LC-MS methods.

Comparison of nano-ESI-MS and LC-MS methodology applied to standards and dilute cell extracts

Standard calibration curves for each amino acid were used to assess method sensitivity, precision and linearity of the LC-MS and NSI methods. Supplementary Table S4 demonstrates that LC-MS was the superior method for each of these criteria.

A THP-1 metabolite extract was diluted to the single-cell-level and analyzed using both nano-ESI- and LC-MS methods. Supplementary Figure S12 shows that more features were detected in the single-cell-level metabolite extract by LC-MS than by nano-ESI-MS. A greater number of features were detected in capillary sampled single cells compared to diluted cell extract in both methods. Similar observations were found by von Gerichten et al. when comparing dilute lipid extracts and single cells.^20^ Supporting Information S13 shows a significant increase in the average number of features detected in single cells in comparison to single-cell-level metabolite extract when analyzed by LC-MS. There was no significant difference between the two matrices when analyzed by nano-ESI-MS.

### Comparison of Nano-ESI-MS and LC-MS Methodology Applied to Standards and Dilute Cell Extracts

Standard calibration curves for each amino acid were used to assess method sensitivity, precision and linearity of the LC-MS and NSI methods. Supplementary Table S4 demonstrates that LC-MS was the superior method for each of these criteria.

A THP-1 metabolite extract was diluted to the single-cell-level and analyzed using both nano-ESI- and LC-MS methods. Supplementary Figure S12 shows that more features were detected in the single-cell-level metabolite extract by LC-MS than by nano-ESI-MS. A greater number of features were detected in capillary sampled single cells compared to diluted cell extract in both methods. Similar observations were found by von Gerichten et al. when comparing dilute lipid extracts and single cells.^20^ Supporting Information S13 shows a significant increase in the average number of features detected in single cells in comparison to single-cell-level metabolite extract when analyzed by LC-MS. There was no significant difference between the two matrices when analyzed by nano-ESI-MS.

### Single-Cell Sampling and Analysis

To compare the ability of the nano-ESI- and LC-MS methods to analyze metabolites from a BCG macrophage infection model, 20 infected cells and 20 unexposed control cells were sampled for each method using the SS2000. To confirm that individual cells were successfully sampled, video footage was captured during sampling and *Z*-stack imaging was performed before and after cell aspiration (Figure 1). Infected cells were identified by observing mCherry fluorescent BCG using confocal microscopy, as seen in Figure 1C.

**Figure 1:**
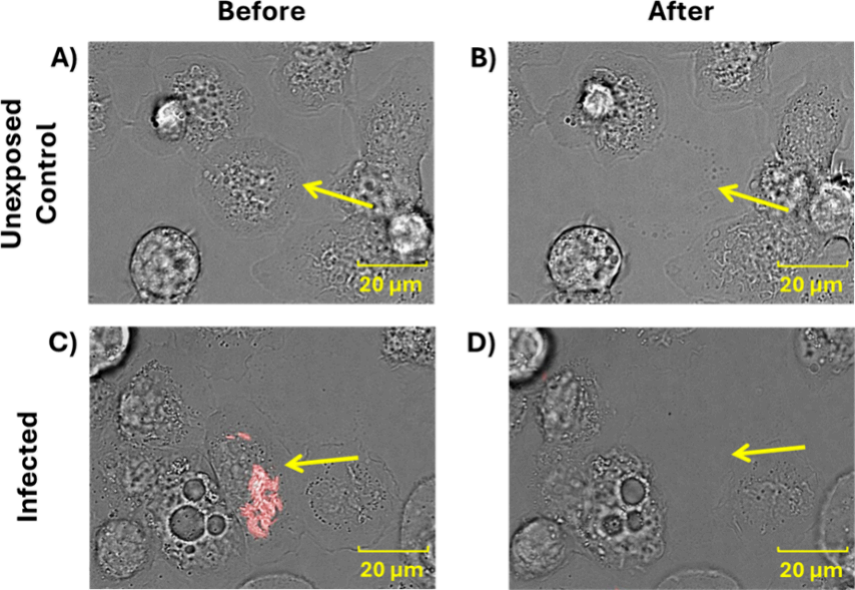
Unexposed control THP-1 macrophages before (A) and after (B) sampling. Red-fluorescent BCG infected cell before (C) and after (D) sampling.

Of the 80 cells sampled, 20 BCG infected and 18 unexposed control macrophages were successfully analyzed using nano-ESI- and LC-MS respectively. Labeled internal standard and unlabeled amino acids were monitored in single cells and PBS blanks to confirm the sample had eluted from the capillary tip (Supplementary Figures S14 and S15). Figure 2 compares the untargeted analysis data for both methods, focusing on the total number of features detected. The figure shows that nano-ESI-MS detected fewer *m*/*z* values, annotated formulas, and named compounds than LC-MS in single-cells (Figure 2A). We propose that this may be due to ion suppression, reducing the intensities of ions below the limit of detection. 203 annotated compounds were detected using both LC- and nano-ESI-MS (Figure 2B). This accounts for the majority (59%) of annotated compounds detected using nano-ESI-MS, with 141 compounds (41%) identified as unique annotations. In contrast, a greater proportion of annotations detected by LC-MS were unique (74%). Nevertheless, nano-ESI- and LC-MS were able to detect a broad spectrum of metabolite classes from the HMDB, with LC-MS consistently detecting more compounds in each class in the single cells measured (Figure 2C). The overlapping classes detected in the two methods indicate they are both able to capture a wide metabolic profile. LC-MS is more suited to detecting larger, more complex metabolites in single cells, such as fatty acyls, while nano-ESI-MS is superior at detecting smaller molecules, such as diazines and azoles. This additional biochemical context aids in interpreting metabolite detection and guides the choice of analytical method best suited for specific single-cell biological questions.

**Figure 2:**
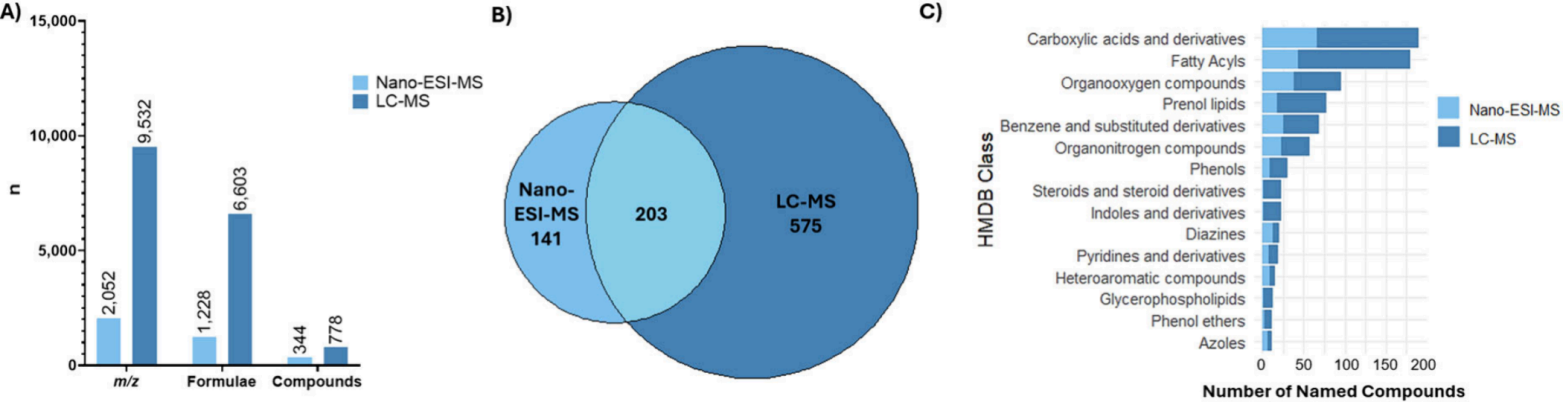
Summary of features detected in single BCG infected and unexposed control THP-1 macrophages by nano-ESI- and LC-MS. (A) Number of total features subcategorized into those that can be annotated with formulas and/or compound names. (B) Overlap of number of annotated named compounds detected by each method. (C) Top 15 HMDB classes in which the greatest number of annotated named compounds were detected.

To screen for differences in features detected in infected and unexposed control macrophages, multivariate analysis was carried out. Figure 3A,D shows partial least-squares-discriminant analysis (PLS-DA) for nano-ESI-MS and LC-MS data. PBS blanks, infected macrophages and control-unexposed cells are more clearly separated using the LC-MS method. While the two models differed substantially in the proportion of class-associated variance explained (Figure 3A, R^2^ = 0.09; Figure 3D, R^2^ = 0.75), both exhibited limited predictive performance (nano-ESI-MS Q^2^=-0.06; LC-MS Q^2^=0.05), assessed by leave one out cross validation (LOOCV), Supplementary Tables S5 and S6. This is consistent with the use of PLS-DA as an exploratory tool to visualize multivariate trends in heterogeneous single-cell data classification. The heatmaps in Figure 3B,E show the intensity of the top 50 features (those determined by ANOVA to be significantly different between the sample groups). The features detected using nano-ESI-MS (Figure 3B) show considerable variability compared to those detected by LC-MS (Figure 3E) and the delineation between blanks, control and infected cells is less clear. In contrast, the LC-MS data shows a cluster of metabolite features which are enriched in infected cells, and there is a clear distinction between cells and blanks. Figure 3C,F display volcano plots for control and infected cells by nano-ESI-MS and LC-MS. For nano-ESI-MS, the majority of significant features were enriched in BCG-infected macrophages, whereas LC-MS detected a more balanced distribution of enriched and depleted features between the two cellular groups, overall detecting a greater number of features.

**Figure 3:**
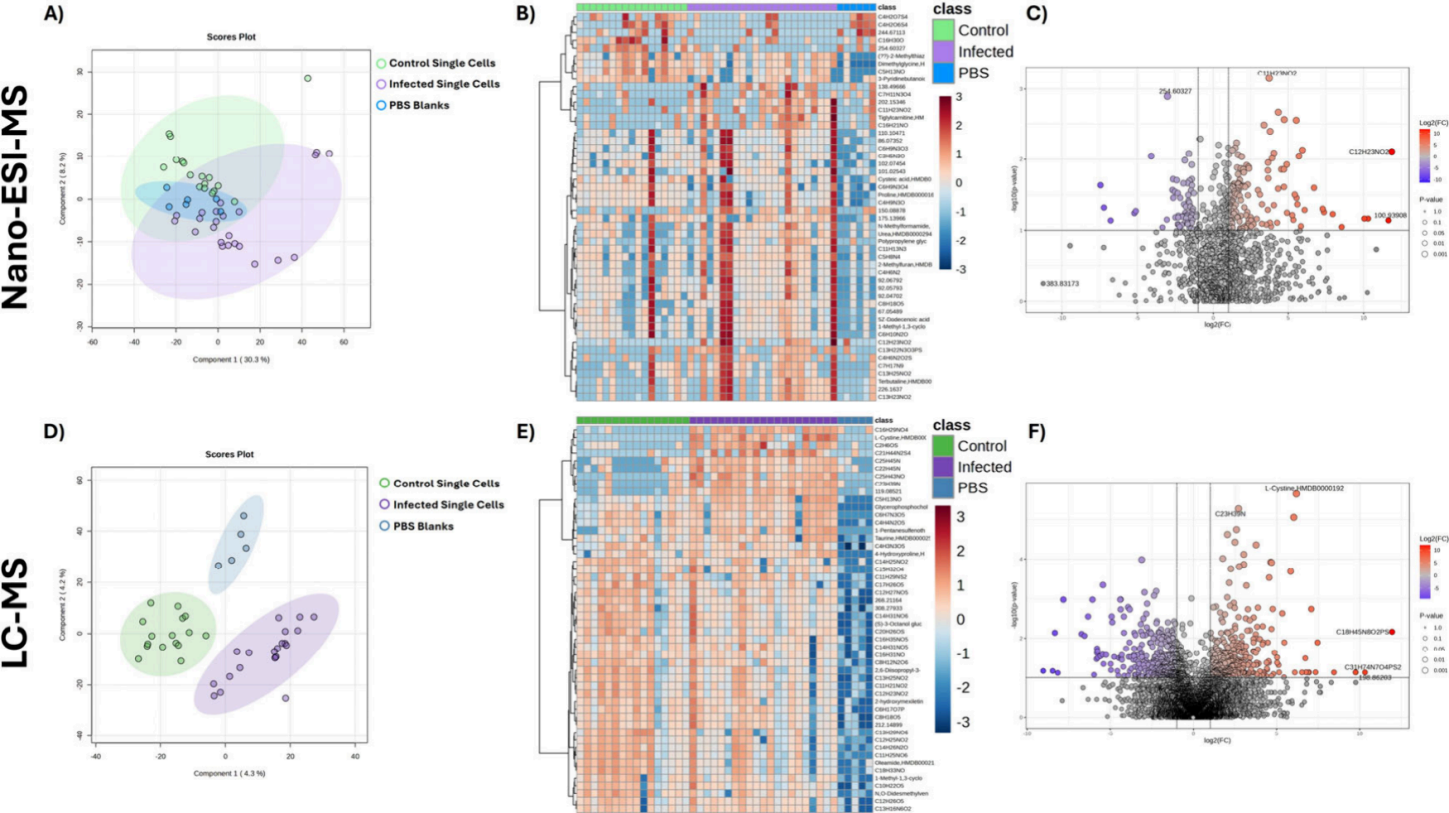
Nano-ESI- and LC-MS untargeted analysis results including named compound and formula annotations and *m*/*z* features. PLS-DA of all features for nano-ESI (A) and LC-MS (D). Heatmaps of top 50 ANOVA significant features detected in unexposed control and infected single macrophages compared to PBS blanks for nano-ESI (B) and LC-MS (E). Clustering of samples was supervised. Volcano plot comparing Wilcoxon *t* test significant features in infected/unexposed control single macrophages for nano-ESI (C) and LC-MS (F).

Taurine and cystine were both significantly enriched in BCG infected macrophages detected by LC-MS (Figure 3E and F). It is well-documented that taurine, a sulfonic acid, plays a role in modulating inflammatory mediators and controlling macrophage M1 polarization.^49–52^ Taurine is metabolically linked to cysteine and other sulfur-containing compounds, such as methionine, which play a crucial role in mycobacterial infection.^53–55^ The parallel enrichment of cystine, the oxidized disulfide of cysteine, is indicative of increased cystine import and intracellular reduction, consistent with redox adaptation of macrophages to infection-induced oxidative stress.^56^

Figure 4A shows the structural formulas of methionine, cysteine, cystine and taurine. The enrichment of sulfur metabolism associated genes has been consistently linked to oxidative stress within the macrophage upon Mtb infection.^55,57^ In concordance with this, BCG infected single macrophages analyzed by LC-MS exhibited significantly elevated levels of methionine, cysteine, cystine, and taurine, features which are consistent with redox buffering and antioxidant defense mechanisms (Figure 4C).^58,59^ However, taurine was not detected by nano-ESI-MS and, after multiple Mann-Whitney U *t* tests, methionine, cysteine and cystine were found to be insignificant (Figure 4B).

**Figure 4:**
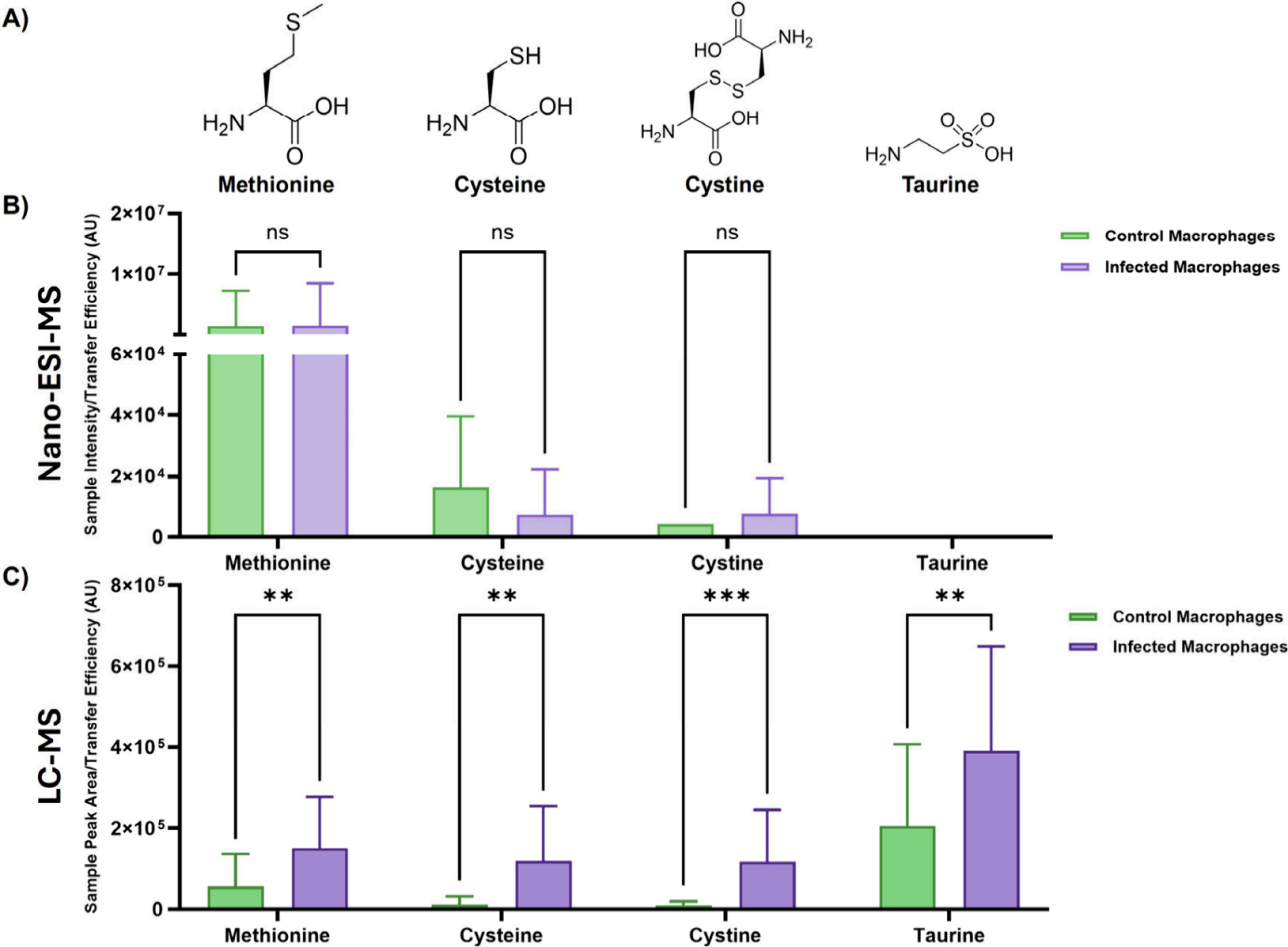
A) Structural formulas of methionine, cysteine, cystine and taurine. Transfer efficiency normalized peak area of methionine, cysteine, cystine and taurine in unexposed control (green) and BCG infected (purple) single THP-1 derived macrophages for nano-ESI (B) and LC-MS (C). Multiple Mann-Whitney U *t* tests with Holm-Šídák correction for multiple comparisons were performed for each analyte.

Supplementary Table S7 lists the named compound annotations that were both detected above 10x S/N by the two methods in untargeted analysis and varied significantly (Wilcoxon *t* test) between unexposed control and infected single cells. The LC-MS method revealed 95 metabolite features that varied between control and infected cells, while the nano-ESI-MS method found 35, with little overlap between the two methods. This is presumably due to the differences in sensitivity and precision between the two methods to various analyte classes, as displayed in Figure 2C. Only one feature was determined by both methods to be significantly enriched in infected cells. This low concordance in directional metabolic changes at the single-cell level suggests that the techniques are more complementary rather than interchangeable.

The ability to spatially sample macrophages containing a pathogen removes the possibility of uninfected cells contributing to these results and potentially diluting the true metabolite profile. This also provides the capability for future work to distinguish between direct pathogen-host interaction and so-called ‘bystander’ immune effects, in which adjacent infected and uninfected cells affect each other’s metabolism.

Single THP-1 macrophages were isolated using the Yokogawa SS2000, a newly commercialized instrument that enables capillary-based sampling of live cells under controlled temperature, CO_2_, and humidity, with confocal microscopy visualization. This approach allows spatial analysis of living cells to address important unresolved questions in cell biology, such as whether bystander effects protect cells from microbial infection. In principle, cell sampling can be preprogrammed, with each single cell taking approximately 5 min to sample and so, although the cell sampling is not as fast as microfluidics methods, the rate-limiting step is currently the mass spectrometry.

Direct, offline nano-ESI-MS offers clear advantages in terms of reduction in time and costs as there are no transfer steps and each sample can be analyzed within 2 min. However, these advantages are offset by the lack of automation (each tip must be manually loaded to be analyzed) and the capillary tips frequently clogging, which leads to failed sample analysis. Approaches are available to automate nano-ESI-MS data acquisition, however, our results have demonstrated that only limited metabolite profiles at the single-cell level are achieved compared with LC-MS. While dedicated processing pipelines for direct-infusion data are emerging, robust open-access workflows remain limited, which presently constrains routine implementation. Consequently, nano-ESI-MS is currently best positioned as a shotgun technique to quickly screen capillary-sampled single cells.

LC-MS is often criticized in single-cell work for requiring dilution associated with chromatographic separation, which many assume will compromise sensitivity to low abundant analytes. Additionally, the cell must be transferred from the capillary to a vial, with the potential for analyte loss in transfer. In contrast, our results show that even with the dilution of analytical flow rates, separation enhances sensitivity by reducing ion suppression and improving metabolite coverage, and permits isomeric resolution essential for confident metabolite annotation. The limitations include longer analysis times and higher cost per sample compared to nano-ESI-MS. Additionally, the process of transferring the cell from the tip to the vial is not automated. Nevertheless, LC-MS delivers high quality data, making it well suited for comprehensive metabolic profiling of single cells. A further advantage is that the LC-MS data analysis workflows are far more well-established and routine, with many open-access tools available to support both untargeted and targeted metabolomics. Development of fully validated targeted methods suitable for clinical or regulatory use, automation of transferring cells from tips to vials and using nano flow rates to boost sensitivity in the LC-MS method are scope for future studies in this area. Table 1. compares the advantages and disadvantages of nano-ESI-MS and LC-MS techniques. Taken together, both nano-ESI-MS and LC-MS offer distinct strengths, making them well suited to different experimental contexts. Nano-ESI-MS provides speed and minimal handling, making it ideal for exploratory or high-throughput single-cell screening, whereas LC-MS delivers higher sensitivity and greater metabolite coverage, supporting comprehensive single-cell metabolomics.

**Table 1:** Advantages and Limitations of Nano-ESI-MS and LC-MS as Techniques for Single-Cell Metabolomics Analysis

Nano-ESI		LC-MS	
Advantages	Limitations	Advantages	Limitations
Higher throughput (2 min/cell)	Inferior coverage of analytes compared with LC-MS	Good coverage of analytes covering multiple metabolite classes	Poorer throughput compared with nanospray (15 min/cell)
Fewer consumables	Difficulty annotating peaks	Peak annotation using widely available software	Uses more consumables and requires the cell to be transferred from a tip to a vial
	Poorer detection limits compared to LC-MS	Good detection limits compared with nano-ESI-MS	
	Clogging of tip can make it difficult to spray some cells	Good separation of infected cells, controls and blanks based on metabolite profile	

## Conclusion

In this study, we reported the first single-cell comparison of nano-ESI-MS and LC-MS for metabolomics, providing complementary strategies for the detection of amino acids and hydrophilic metabolites. LC-MS offered the broadest metabolite coverage and superior detection limits, likely due to reduced ion suppression, despite significant dilution. Nano-ESI-MS required minimal sample handling and achieved ∼7.5-fold faster analysis, making it a cost-effective shotgun approach for rapid profiling. These distinct platforms support strategic experimental design, enabling analysts to balance speed, depth, and practicality.

The LC-MS approach uncovered infection-driven reprogramming of sulfur metabolism, characterized by the increased levels of methionine, cysteine, cystine, and taurine. These findings establish single-cell metabolomics as a powerful means of resolving infection-driven heterogeneity, with future work aimed at distinguishing direct host-pathogen interactions from bystander immune effects. The spatial component of this framework provides a foundation for advancing disease biology, accelerating drug discovery, and improving clinical diagnostics.

## Supplementary Material



## Notes

Labeled and nonlabeled amino acid standard concentrations, LC-MS and nano-ESI-MS optimization, comparison of solvent brands, limits of detection and quantification for amino acids, extracted ion chromatograms, leave one out cross validation, and a list of significant named compounds detected in both methods (PDF)
