# Supplementary material for: Comparison of Liquid Chromatography- and Nano-Electrospray Ionization-Mass Spectrometry Approaches for Single-Cell Metabolomics
Source: Anal Chem. 2026 Mar 18;98(12):8956–65. doi: 10.1021/acs.analchem.5c06318 (PMC13044881; doi:10.1021/acs.analchem.5c06318)
Supplement: Supplementary file 1 [file ac5c06318_si_001.pdf]

Supporting Information

Comparison Of Liquid Chromatography- and Nano-Electrospray Ionization-Mass Spectrometry Approaches for Single-Cell Metabolomics.

Abigail Cook[1,2], Claire Davison[2], Jordan Pascoe[3], Harpreet Atwal[1], George Mayson[3], Ahmed Ali[4], Dany JV Beste[3], Melanie Bailey[2,1]*

[1] Faculty of Engineering and Physical Sciences, University of Surrey, Guildford, GU2 7XH, UK
[2] Department of Infectious Diseases, King's College London, Guy's Hospital, London, SE1 9RT, UK
[3] Faculty of Health and Medical Sciences, University of Surrey, Guildford, GU2 7XH, UK
[4] Metabolomics & Analytics Centre, LACDR, Leiden University, 2300 RA Leiden, Netherlands
*Email: melanie.j.bailey@kcl.ac.uk

Table of Contents

Table S1. Deuterated analytes and respective concentrations in stock D-aa standard. S3
Table S2. Analytes present in non-labelled aa standard. S3
Table S3. Concentrations of amino acids in each calibration non-labelled aa standard. S4
Figure S1. Comparing signal to noise ratio of cell extract at 50-, 10- and 1-cell concentration of original base method to optimized method using LC-MS. Non-parametric Wilcoxon T-Test. N = 3. 4
Figure S2. Total number of m/z, formulae and named compounds detected in single-cell-level extract analyzed by Base and Optimized Methods using LC-MS. N = 3. ....S4
Figure S3. Comparison of three solvent brands: amino acid signal/noise normalized to respective deuterated amino acids using LC-MS. Multiple Mann-Whitney U t-tests with Holm-Šídák correction for multiple comparisons. N = 3. ....S5
Figure S4. Percentage recovery of deuterated amino acids either eluted from capillary tip to vial with 10 µL mobile phase added on top (Wash) or into a vial preloaded with 10 µL mobile phase (No Wash) using LC-MS. N = 5. ....S5
Figure S5. Comparison of internal standard normalized peak areas of single-cell-level metabolite extract analyzed at 70,000 and 140,000 resolution settings for LC-MS. Multiple Mann-Whitney U t-tests with Holm-Šídák correction for multiple comparisons. N=6. ....S6
Figure S6. Total number of *m/z*, formulae and named compounds detected for 70,000 and 140,000 resolutions applied to single-cell-level metabolite extract using LC-MS. N=6. ....S6
Figure S7. Number of m/z, formulae and named compounds detected for 70,000 and 140,000 resolutions applied to single-cell-level metabolite extract using LC-MS. Multiple Mann-Whitney U t-tests with Holm-Šídák correction for multiple comparisons. N=6. ....S7
Figure S8. Optimization of nanospray emitter position by spraying single-cell-level metabolite extract and manipulating *x, y* and *z* loci. The intensity of total ion current was assessed. Black = start position; Red = move tip up the *z*-plane, closer to the detector; Green = move along *x*-plane, left; Purple = move down *y*-plane; Yellow = move up *y*-plane, back to original y-position. S7
Figure S9. Internal standard normalized amino acid peak intensity responses to changing resolution between 70,000, 140,0000 and 280,000 in single-cell-level metabolite extract using nano-ESI-MS. Multiple Mann-Whitney U t-tests with Holm-Šídák correction for multiple comparisons. N=6. ....S7
Figure S10. Total number of *m/z*, formulae and named compounds detected in single-cell level metabolite extract using nano-ESI-MS at 70,000, 140,000 and 280,000 resolutions. N=6. ....S8

Figure S11. Average number of *m/z*, formulae and named compounds detected in single-cell level metabolite extract using nano-ESI-MS at 70,000, 140,000 and 280,000 resolutions. Multiple Mann-Whitney U t-tests with Holm-Šídák correction for multiple comparisons. N=6. .....S8
Table S4. Limits of detection and quantification, percentage relative standard deviation and linearity of amino acids in calibration standards for both nano-ESI-MS and LC-MS. ND = Not detected. S9
Figure S14. LC-MS extracted ion chromatograms of unlabeled and deuterated amino acids in a PBS blank (A and B), a control unexposed single cell (C and D), and an infected single cell (E and F). Each color represents an unlabeled or labeled amino acid. Deuterated amino acids were added to the PBS blanks as an internal standard...S11
Figure S15. Nano-ESI-MS extracted ion chromatograms of unlabeled and deuterated amino acids in a PBS blank (A and B), a control unexposed single cell (C and D), and an infected single cell (E and F). Each color represents an unlabeled or labeled amino acid. Deuterated amino acids were added to the PBS blank as an internal standard. S12
Table S5. Leave one out cross validation (LOOCV) results for PLS-DA of all features in single infected and control cells and PBS blanks, analyzed by nano-ESI-MS. S13
Table S6. Leave one out cross validation (LOOCV) results for PLS-DA of all features in single infected and control cells and PBS blanks, analyzed by LC-MS. S13
Table S7. Comparison of Wilcoxon t-test significant named compounds detected in single cells by nano-ESI-MS or LC-MS. Green reflects a significantly higher intensity (log2FC > 0) and red reflects a significantly lower intensity (log2FC < 0) within infected single cells compared to control unexposed single cells. Blank reflects the named compound was not statistically significant or was not detected. S14

Table S1. Deuterated analytes and respective concentrations in stock D-aa standard.

Analyte	Conc (µM)
Alanine-d4	164
Arginine-d7	48
Aspartic Acid-d3	20
Cystine-d4	24
Glutamic Acid-d5	64
Glutamine-d5	222
Glycine-d2	152
Histidine-d5	50
Isoleucine-d10	39
Leucine-d10	85
Lysine-d8	70
Methionine-d8	24
Phenylalanine-d8	44
Proline-d7	84
Serine-d3	60
Threonine-d5	81
Tryptophan-d8	31
Tyrosine-d7	45
Valine-d8	156

Table S2. Analytes present in non-labelled aa standard.

Analyte
L-Alanine
L-Arginine
L-Aspartic acid
L-Cystine
L-Glutamic acid
Glycine
L-Histidine
L-Isoleucine
L-Leucine
L-Lysine
L-Methionine
L-Phenylalanine
L-Proline
L-Serine
L-Threonine
L-Valine

Table S3. Concentrations of amino acids in each calibration non-labelled aa standard.

Calibration number	Concentration (nM)
1	0.5000
2	0.4378
3	0.3757
4	0.3135
5	0.2514
6	0.1892
7	0.1271
8	0.0649
9	0.0028

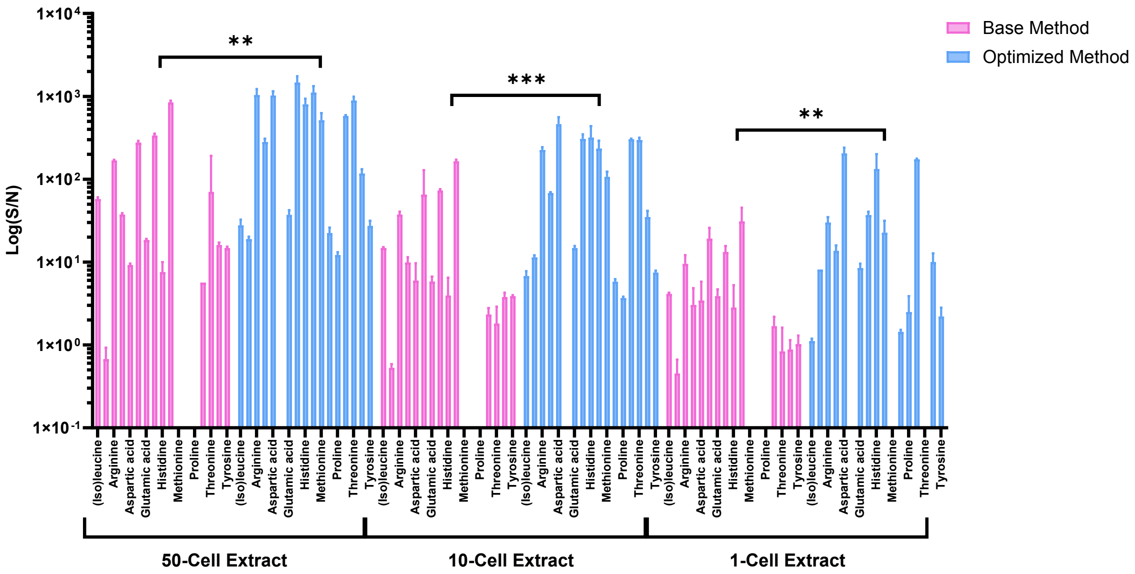

Figure S1. Comparing signal to noise ratio of cell extract at 50-, 10- and 1-cell concentration of original base method to optimized method using LC-MS. Non-parametric Wilcoxon T-Test. N = 3.

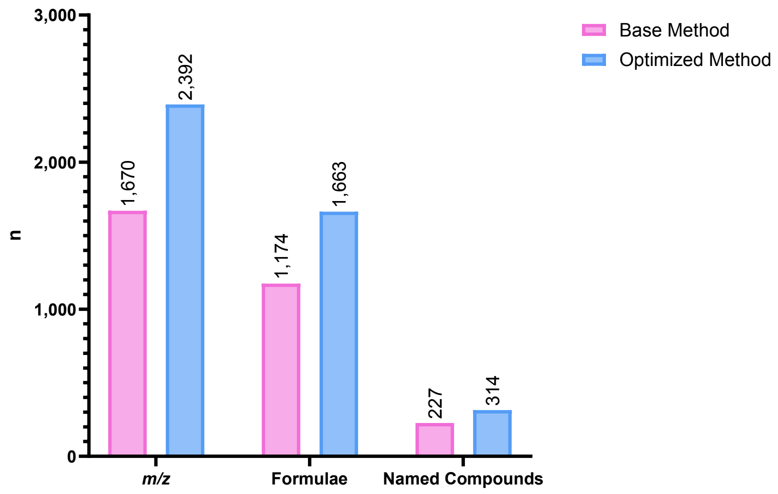

Figure S2. Total number of m/z, formulae and named compounds detected in single-cell-level extract analyzed by Base and Optimized Methods using LC-MS. N = 3.

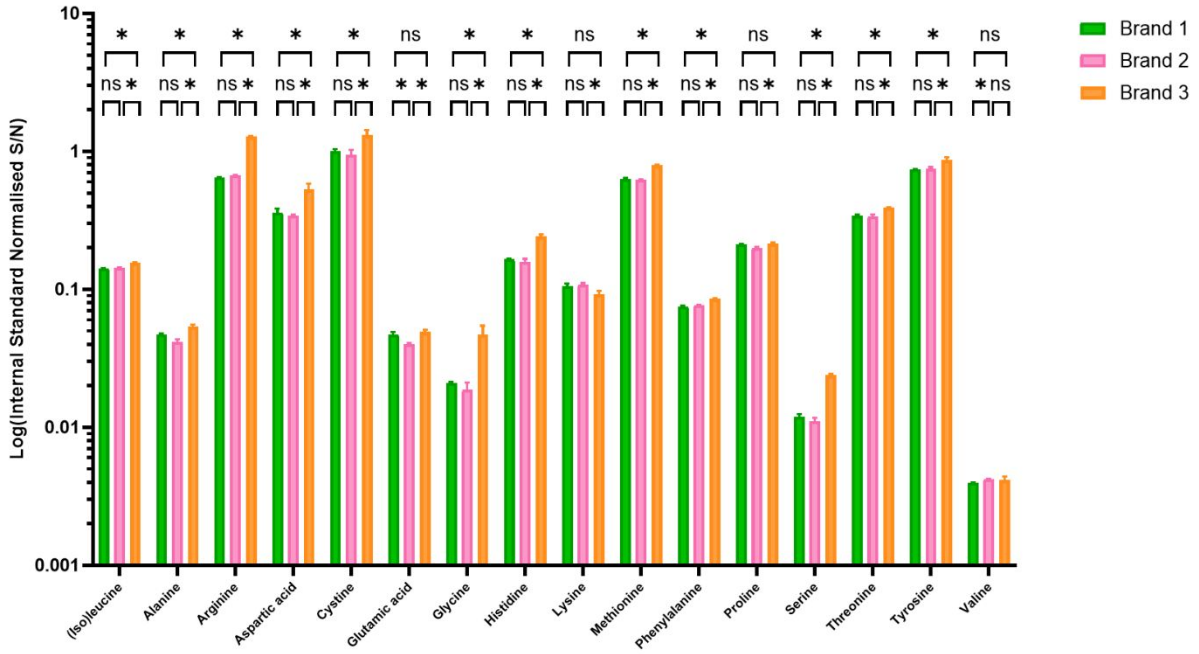

Figure S3. Comparison of three solvent brands: amino acid signal/noise normalized to respective deuterated amino acids using LC-MS. Multiple Mann-Whitney U t-tests with Holm-Šídák correction for multiple comparisons. N = 3.

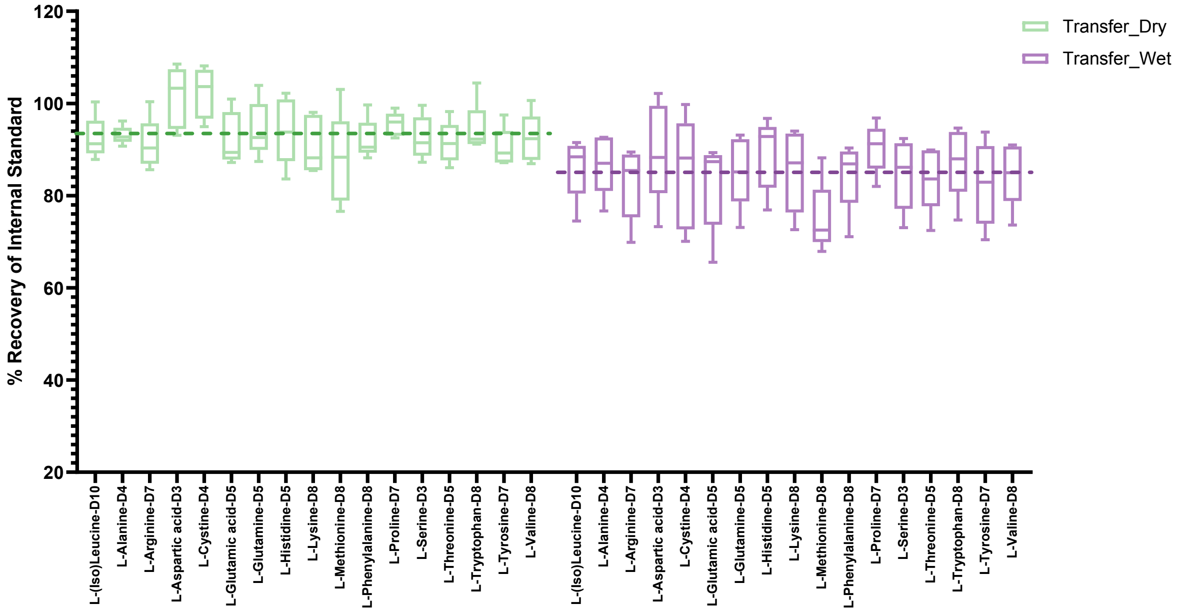

Figure S4. Percentage recovery of deuterated amino acids either eluted from capillary tip to vial with 10 µL mobile phase added on top (Wash) or into a vial preloaded with 10 µL mobile phase (No Wash) using LC-MS. N = 5.

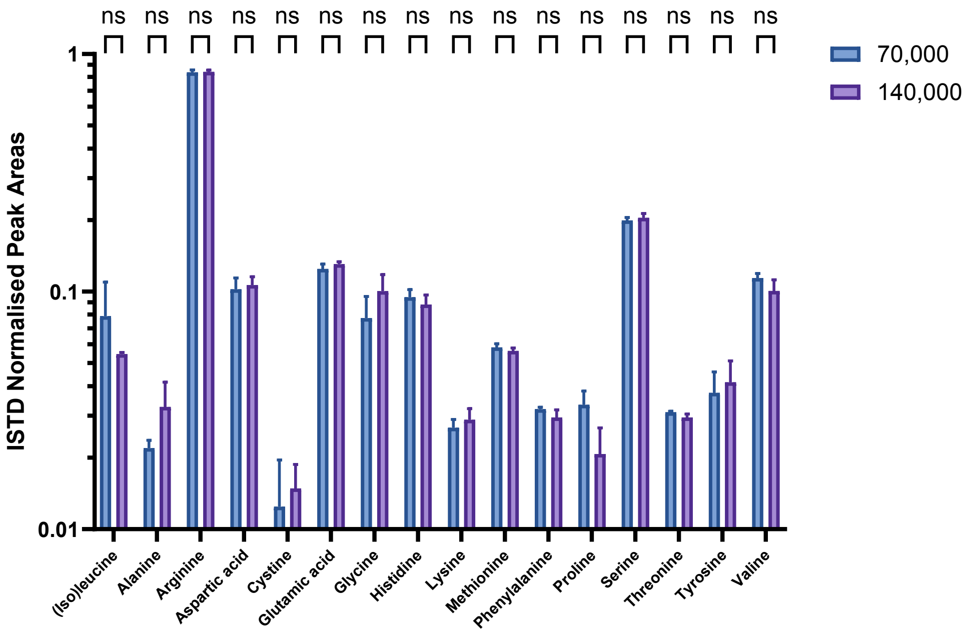

Figure S5. Comparison of internal standard normalized peak areas of single-cell-level metabolite extract analyzed at 70,000 and 140,000 resolution settings for LC-MS. Multiple Mann-Whitney U t-tests with Holm-Šídák correction for multiple comparisons. N=6.

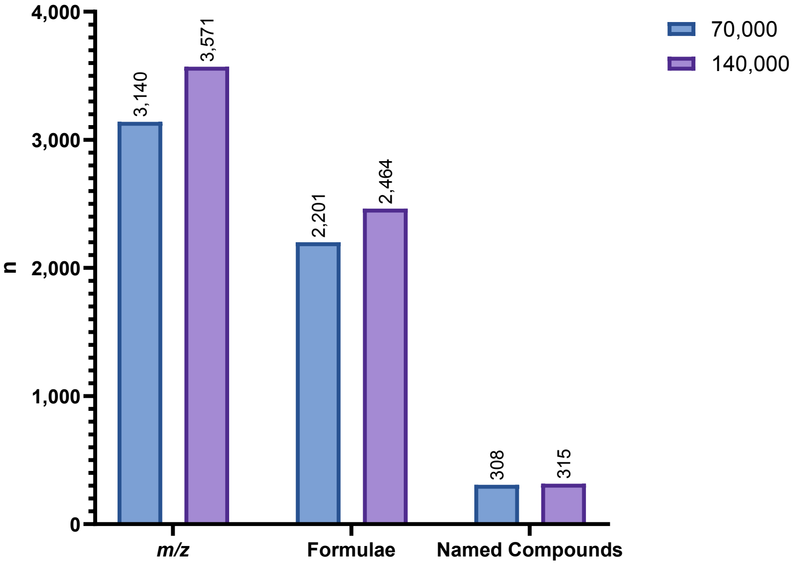

Figure S6. Total number of *m/z*, formulae and named compounds detected for 70,000 and 140,000 resolutions applied to single-cell-level metabolite extract using LC-MS. N=6.

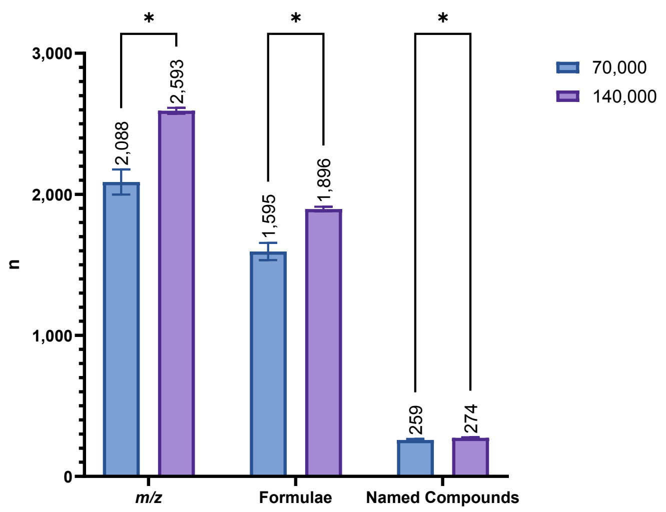

Figure S7. Number of m/z, formulae and named compounds detected for 70,000 and 140,000 resolutions applied to single-cell-level metabolite extract using LC-MS. Multiple Mann-Whitney U t-tests with Holm-Šídák correction for multiple comparisons. N=6.

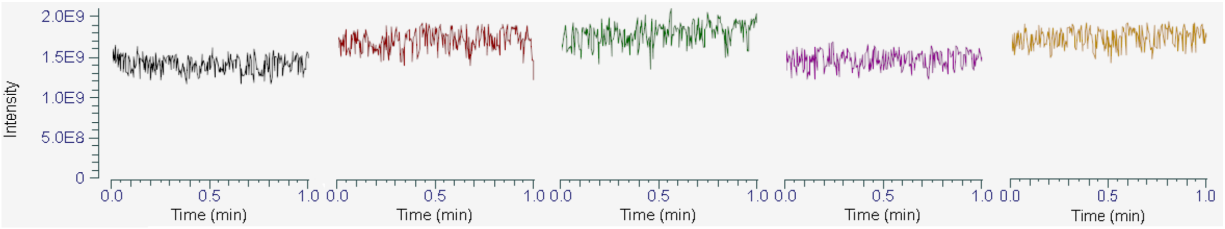

Figure S8. Optimization of nanospray emitter position by spraying single-cell-level metabolite extract and manipulating *x, y* and *z* loci. The intensity of total ion current was assessed. Black = start position; Red = move tip up the *z*-plane, closer to the detector; Green = move along *x*-plane, left; Purple = move down *y*-plane; Yellow = move up *y*-plane, back to original y-position.

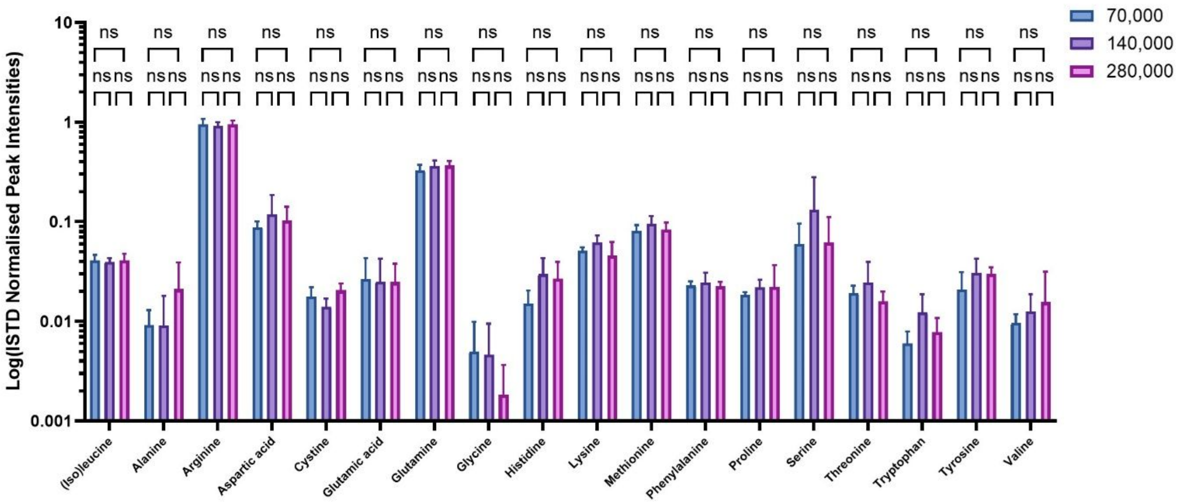

Figure S9. Internal standard normalized amino acid peak intensity responses to changing resolution between 70,000, 140,0000 and 280,000 in single-cell-level metabolite extract using nano-ESI-MS. Multiple Mann-Whitney U t-tests with Holm-Šídák correction for multiple comparisons. N=6.

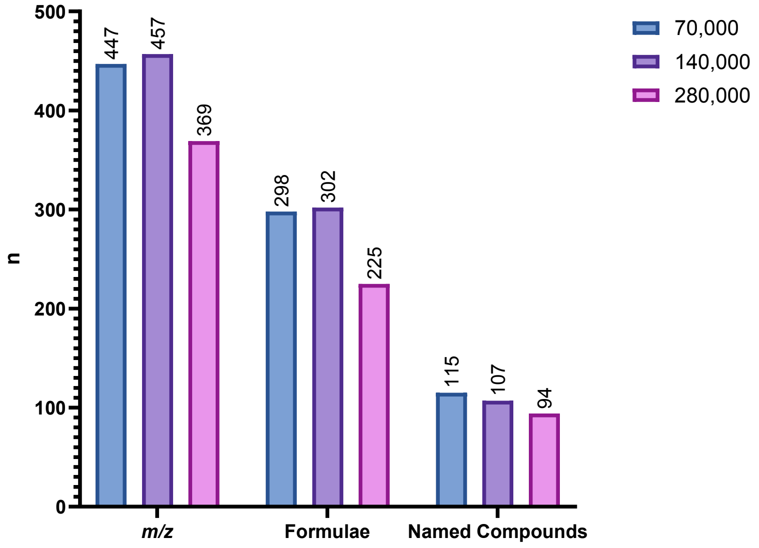

Figure S10. Total number of *m/z*, formulae and named compounds detected in single-cell level metabolite extract using nano-ESI-MS at 70,000, 140,000 and 280,000 resolutions. N=6.

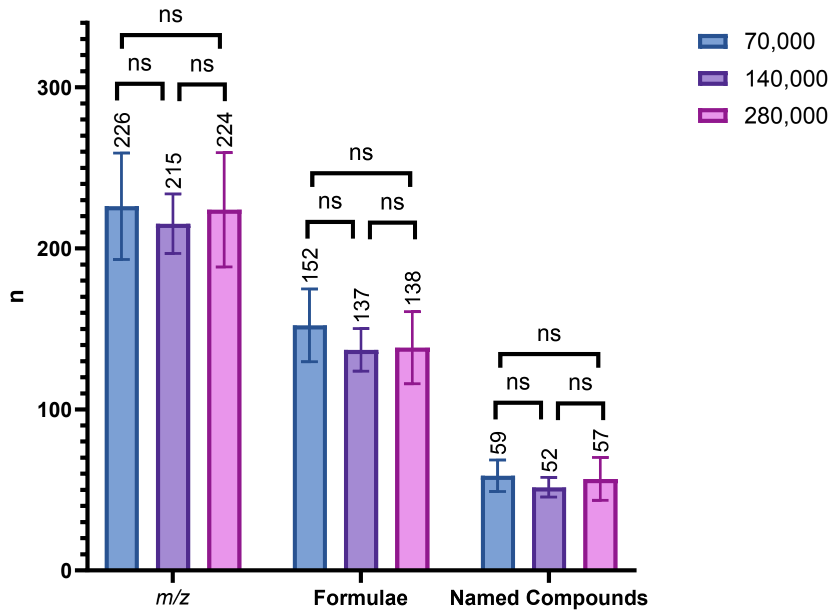

Figure S11. Average number of *m/z*, formulae and named compounds detected in single-cell level metabolite extract using nano-ESI-MS at 70,000, 140,000 and 280,000 resolutions. Multiple Mann-Whitney U t-tests with Holm-Šídák correction for multiple comparisons. N=6.

Table S4. Limits of detection and quantification, percentage relative standard deviation and linearity of amino acids in calibration standards for both nano-ESI-MS and LC-MS. ND = Not detected.

	Nano-ESI-MS				LC-MS			
Compound	LOD (nM)	LOQ (nM)	RSD (%)	Linearity (R^2)	LOD (nM)	LOQ (nM)	RSD (%)	Linearity (R^2)
Alanine	0.048	0.144	5.8	0.9834	0.024	0.073	2.2	0.9980
Arginine	0.056	0.170	2.7	0.9895	0.035	0.107	4.2	0.9958
Aspartate	0.216	0.655	8.2	0.8531	0.027	0.081	3.6	0.9976
Cystine	0.031	0.095	3.6	0.9967	0.056	0.171	5.7	0.9866
Glycine	ND	ND	ND	ND	0.043	0.131	4.1	0.9937
Histidine	0.146	0.441	9.0	0.9174	0.019	0.059	5.8	0.9987
(Iso)Leucine	0.061	0.185	4.0	0.9871	0.296	0.896	3.9	0.9913
Lysine	0.119	0.360	2.8	0.9529	0.023	0.070	2.7	0.9982
Methionine	0.026	0.079	5.4	0.9977	0.024	0.073	1.8	0.9980
Phenylalanine	0.045	0.138	5.2	0.9931	0.023	0.071	4.9	0.9982
Proline	0.099	0.301	3.4	0.9665	0.029	0.089	2.2	0.9971
Serine	2.834	8.587	12.2	0.0285	0.029	0.089	3.0	0.9971
Threonine	0.154	0.465	4.2	0.9090	0.027	0.082	2.7	0.9975
Tyrosine	0.069	0.208	2.8	0.9837	0.023	0.071	1.9	0.9982
Valine	0.209	0.634	4.7	0.8667	0.029	0.088	2.0	0.9972

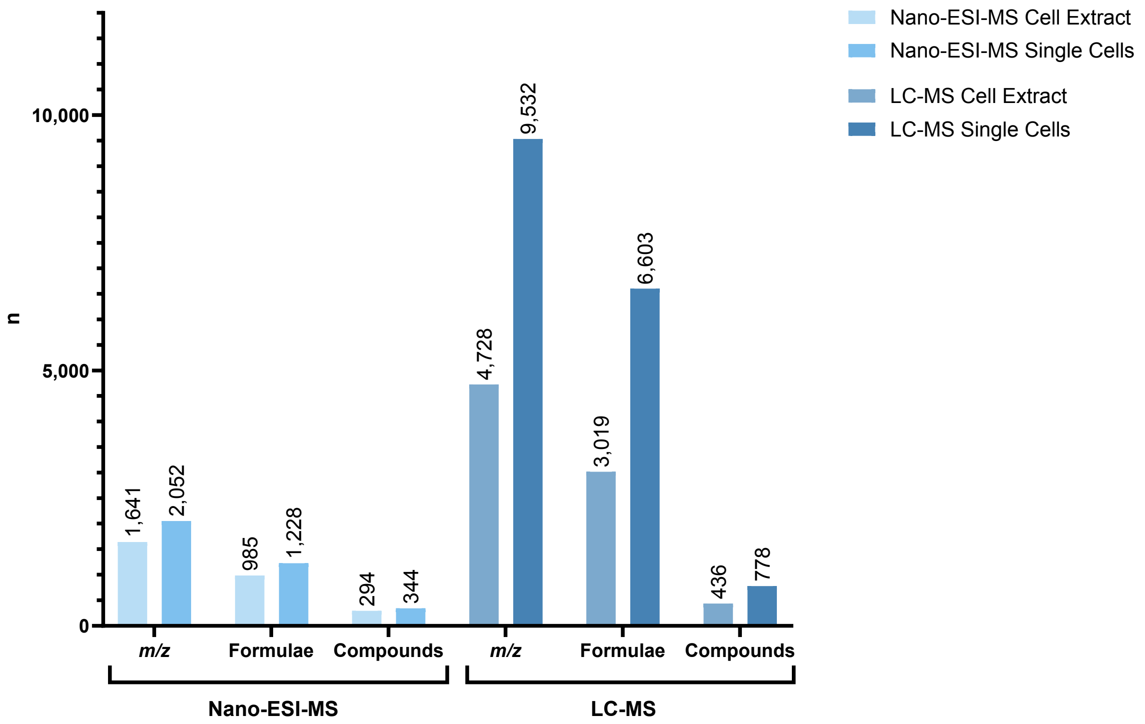

Figure S12. Total number of *m/z*, formulae and named compounds detected in THP-1 metabolite extract (n=4) compared to single THP-1 macrophages (n=40) for both nano-ESI- and LC-MS.

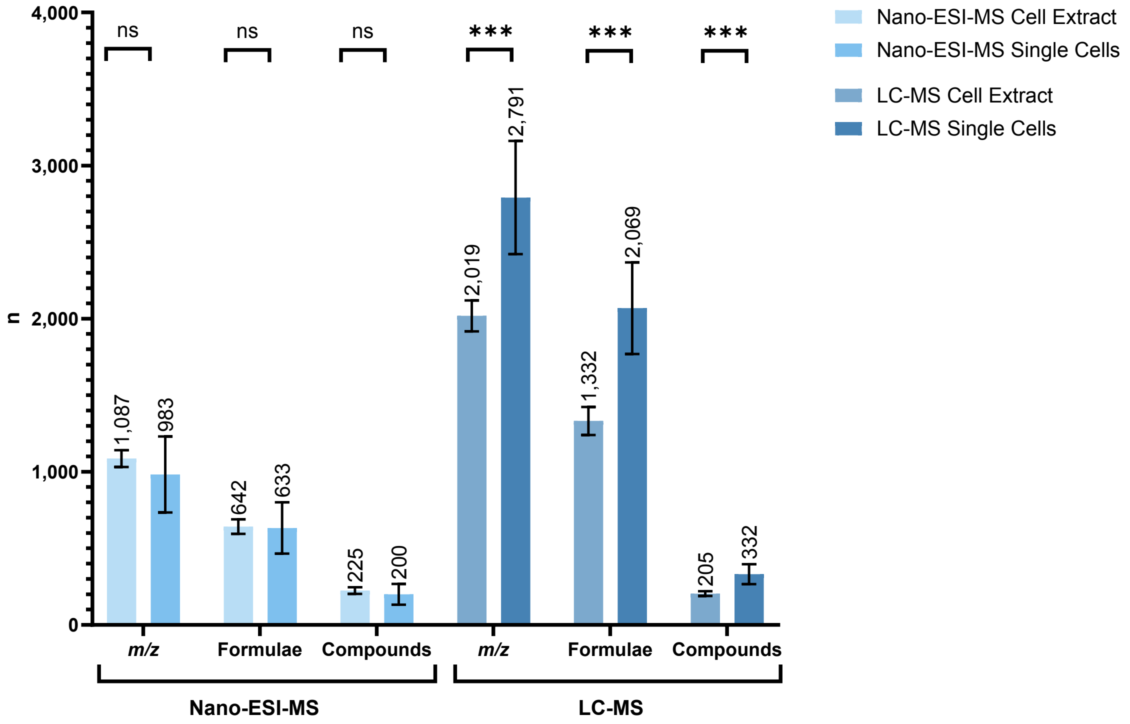

Figure S13. Average number of features detected in THP-1 metabolite extract (n=4) compared to single THP-1 macrophages (n=40) for both nano-ESI- and LC-MS.

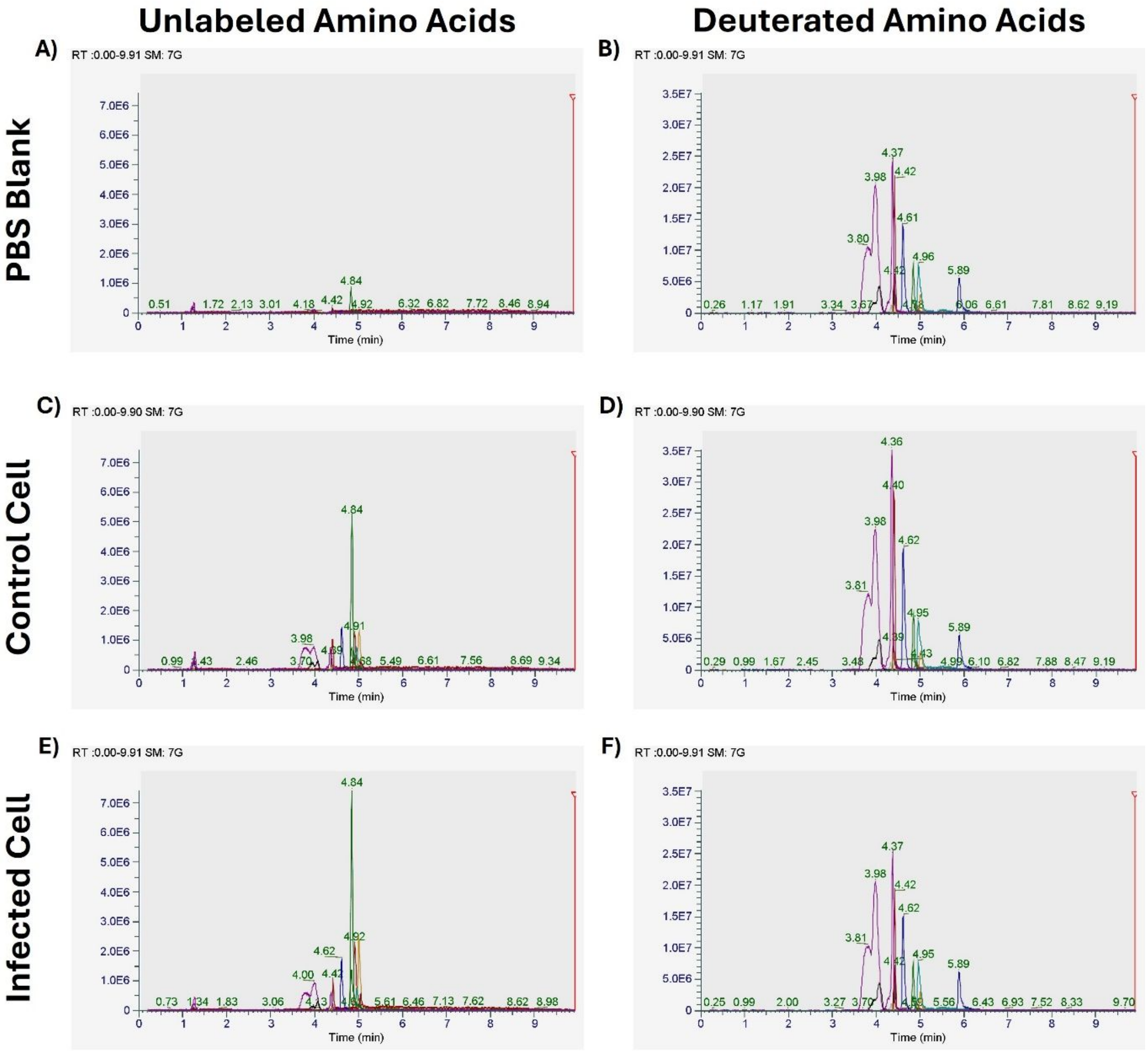

Figure S14. LC-MS extracted ion chromatograms of unlabeled and deuterated amino acids in a PBS blank (A and B), a control unexposed single cell (C and D), and an infected single cell (E and F). Each color represents an unlabeled or labeled amino acid. Deuterated amino acids were added to the PBS blanks as an internal standard.

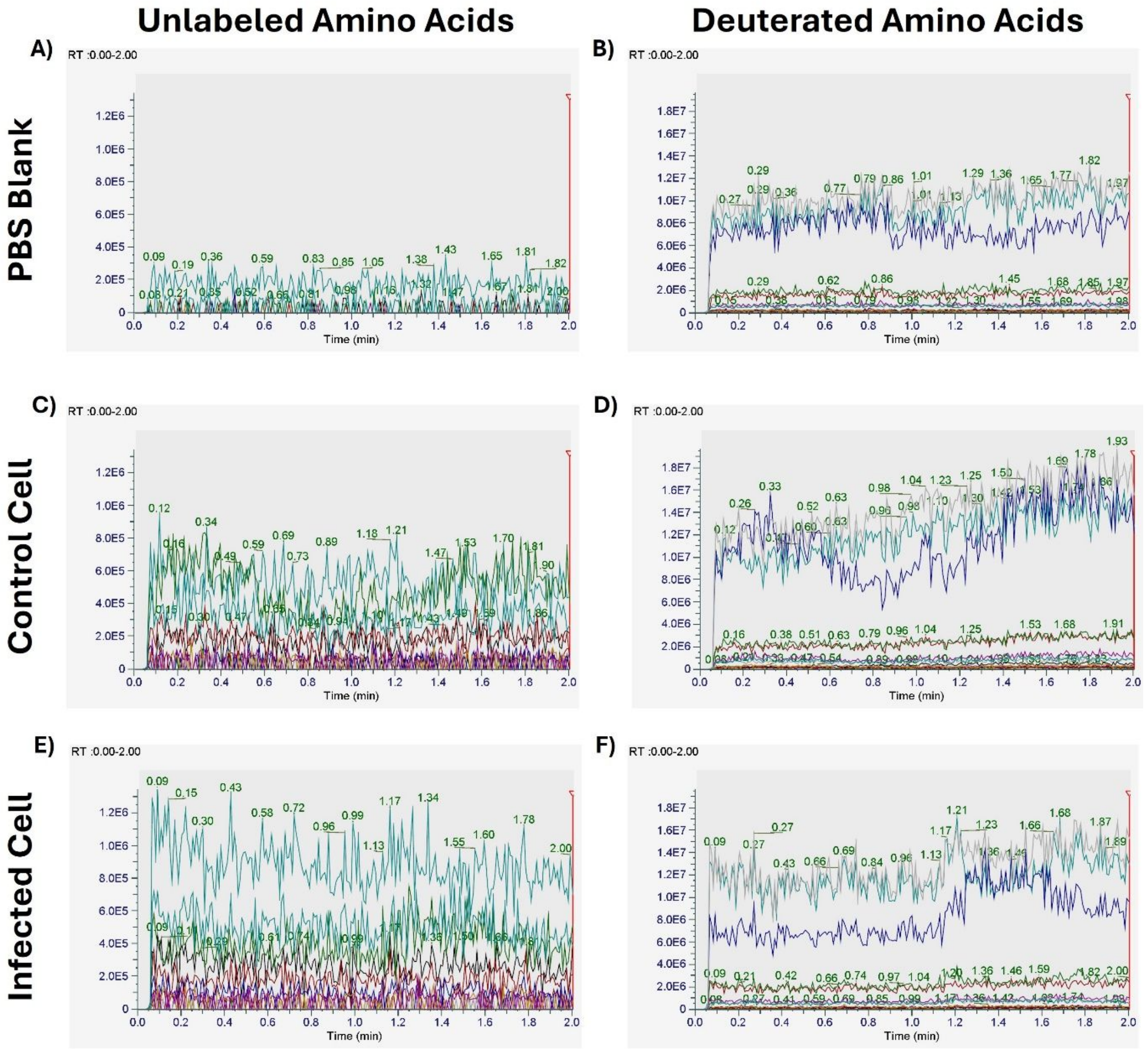

Figure S15. Nano-ESI-MS extracted ion chromatograms of unlabeled and deuterated amino acids in a PBS blank (A and B), a control unexposed single cell (C and D), and an infected single cell (E and F). Each color represents an unlabeled or labeled amino acid. Deuterated amino acids were added to the PBS blank as an internal standard.

Table S5. Leave one out cross validation (LOOCV) results for PLS-DA of all features in single infected and control cells and PBS blanks, analyzed by nano-ESI-MS.

Measure	1 comps	2 comps	3 comps	4 comps	5 comps	6 comps	7 comps	8 comps
Accuracy	0.54348	0.3913	0.56522	0.54348	0.5	0.56522	0.56522	0.58696
R2	0.099253	0.52431	0.76956	0.90833	0.91698	0.96646	0.96858	0.98988
Q2	-0.06872	-0.14115	0.019827	0.14553	0.13906	0.25279	0.20377	0.22057

Table S6. Leave one out cross validation (LOOCV) results for PLS-DA of all features in single infected and control cells and PBS blanks, analyzed by LC-MS.

Measure	1 comps	2 comps	3 comps	4 comps	5 comps	6 comps	7 comps	8 comps
Accuracy	0.52381	0.54762	0.57143	0.61905	0.61905	0.57143	0.61905	0.61905
R2	0.75458	0.75481	0.87285	0.97271	0.97327	0.99198	0.99581	0.99908
Q2	0.057753	0.078067	0.13289	0.1615	0.16456	0.12704	0.14558	0.14374

Table S7. Comparison of Wilcoxon t-test significant named compounds detected in single cells by nano-ESI-MS or LC-MS. Green reflects a significantly higher intensity (log2FC > 0) and red reflects a significantly lower intensity (log2FC < 0) within infected single cells compared to control unexposed single cells. Blank reflects the named compound was not statistically significant or was not detected.

Feature name	Nano-ESI-MS raw p value	Nano-ESI-MS direction	LC-MS raw p value	LC-MS direction
(+)-O-Methylarmepavine,HMDB0030355			1.01E-02	↓
(-)-Nopol,HMDB0030002			2.12E-02	↑
(6R,7S)-6,7-Epoxy-1,3-tetradecadiyne,HMDB0031776	6.20E-02	↑	1.50E-02	↑
(??)-3-Hydroxynonanoic acid,HMDB0031513			8.43E-02	↑
(Acetyloxy)triphenylstannane,HMDB0031789			2.84E-03	↓
(E)-2-Tridecene-4,6,8-triyn-1-ol,HMDB0030931	9.36E-02	↑		
(Z)-3-Methyl-3-decenoic acid,HMDB0031062			2.21E-02	↑
1-(1-Methoxy-1-methylethyl)-4-methylbenzene,HMDB0029653			5.15E-03	↑
1-(2,6,6-Trimethyl-2-cyclohexen-1-yl)-1,6-heptadien-3-one,HMDB0029704			8.71E-02	↑
1-Pentanesulfenothioic acid,HMDB0031160			2.50E-03	↑
1-Phenyl-1-pentanone,HMDB0031208			6.98E-03	↑
2,2,7,7-Tetramethyl-1,6-dioxaspiro[4.4]nona-3,8-diene,HMDB0030007			5.10E-02	↑
2,3-Dimethyl-2-cyclohexen-1-one,HMDB0031414	9.12E-02	↑		
2,4-Di-tert-butylphenol,HMDB0013816	5.45E-02	↑		
2,6-Di-tert-butylbenzoquinone,HMDB0013817			4.19E-02	↑
2-Amino-5-phenylpyridine,HMDB0029747			7.24E-02	↑
2-Dodecenal,HMDB0031020			7.24E-02	↑
2-Ethoxynaphthalene,HMDB0029688			2.30E-02	↑
2-Ethylpyrazine,HMDB0031849	3.65E-02	↑		
2-Methylfuran,HMDB0013749	8.80E-02	↑		
3-(5,6,6-Trimethylbicyclo[2.2.1]hept-1-yl)cyclohexanol,HMDB0031851			9.60E-02	↑
3-Hydroxyhexadecadienoylcarnitine,HMDB0013335			9.02E-02	↓
3-Oxodecanoic acid,HMDB0010724	5.83E-02	↑		
3-Propylidene-1(3H)-isobenzofuranone,HMDB0031845			5.72E-03	↑
4,5-Dihydroorotic acid,HMDB0000528			7.57E-02	↑

Feature name	Nano-ESI-MS raw p value	Nano-ESI-MS direction	LC-MS raw p value	LC-MS direction
4-Hydroxy-alprenolol,HMDB0061122			2.63E-02	↓
4-Hydroxypropofol,HMDB0014018			1.69E-03	↑
4-Methoxycinnamic acid,HMDB0002040			6.41E-02	↓
4-Methyl-1-phenyl-2-pentanone,HMDB0031569			1.77E-02	↑
5-Aminoimidazole,HMDB0003929	6.22E-02	↓		
5-Ethyl-2,3-dimethylpyrazine,HMDB0029727			5.86E-03	↑
5-Hydroxy-L-tryptophan,HMDB0000472			5.79E-02	↑
5-Methyl-2-phenyl-2-hexenal,HMDB0031855			6.37E-02	↑
6-Acetyl-1,2,3,4-tetrahydropyridine,HMDB0030345			2.33E-02	↓
7-Methylguanine,HMDB0000897			9.82E-02	↓
Anethole,HMDB0030837			6.76E-02	↑
Butyrylcarnitine,HMDB0002013			1.82E-02	↑
Cucurbic acid,HMDB0029388			2.56E-02	↑
Cyclohexaneundecanoic acid,HMDB0030997			2.52E-02	↑
D-4-O-Methyl-myo-inositol,HMDB0029915	4.08E-02	↑		
D-Glycero-D-galacto-heptitol,HMDB0033750	4.75E-02	↑		
D-Phenyllactic acid,HMDB0000563			8.45E-02	↑
DL-2-Aminooctanoic acid,HMDB0000991			6.76E-02	↓
Decanoylcarnitine,HMDB0000651			4.66E-02	↑
Diethylphosphate,HMDB0012209			7.93E-03	↑
Dihomo-gamma-linolenic acid,HMDB0002925			2.24E-02	↑
Dihydro-5-(2-octenyl)-2(3H)-furanone,HMDB0030867			1.42E-02	↑
Dihydro-O-methylsterigmatocystin,HMDB0030591			1.02E-02	↑
Dihydrouracil,HMDB0000076	5.17E-02	↑		
Dimethylbenzimidazole,HMDB0003701			3.26E-02	↑
Donepezil metabolite M4,HMDB0013960			7.49E-02	↓
Eicosapentaenoic acid,HMDB0001999			6.46E-02	↑
Eicosapentaenoyl Ethanolamide,HMDB0013649			3.70E-02	↓

Feature name	Nano-ESI-MS raw p value	Nano-ESI-MS direction	LC-MS raw p value	LC-MS direction
Epinephrine,HMDB0000068			1.31E-02	↓
Eremopetasinorol,HMDB0029668			1.86E-03	↑
Farnesol,HMDB0004305			9.32E-02	↑
Fasciculic acid B,HMDB0036438			1.83E-02	↓
Formamidopyrimidine nucleoside triphosphate,HMDB0006822			3.90E-02	↓
Galactitol,HMDB0000107	4.81E-02	↑		
Geranyl acetoacetate,HMDB0038256	2.32E-02	↓		
Glutaminylproline,HMDB0028805	2.59E-02	↑		
Glycerophosphocholine,HMDB0000086			1.66E-03	↑
Goshuyic acid,HMDB0000560			2.84E-02	↑
Hericenone D,HMDB0039139			4.16E-02	↑
Homodihydrojasmone,HMDB0031181			5.68E-02	↑
Hydroxypropionylcarnitine,HMDB0013125	6.93E-03	↑		
Hyperforin,HMDB0030463			6.56E-02	↑
Hypogeic acid,HMDB0002186			1.05E-02	↑
Indoleacetaldehyde,HMDB0001190			3.89E-03	↑
Indoxyl,HMDB0004094			8.45E-02	↑
Isobutylpropylamine,HMDB0031244	8.05E-02	↑		
Isobutyryl-L-carnitine,HMDB0000736			5.49E-02	↓
Isobutyrylglycine,HMDB0000730	3.49E-02	↑		
Kinetensin 1-3,HMDB0012983			1.70E-02	↓
Kynurenic acid,HMDB0000715			4.93E-02	↑
L-Arginine,HMDB0000517			9.93E-03	↑
L-Cystine,HMDB0000192			2.24E-06	↑
MG(0:0/14:1(9Z)/0:0),HMDB0011531			3.10E-02	↑
MG(0:0/16:0/0:0),HMDB0011533			2.67E-02	↑
MG(a-13:0/0:0/0:0)[rac],HMDB0072841			1.15E-02	↑
Methyl (9Z)-8'-oxo-6,8'-diapo-6-carotenoate,HMDB0031979			4.47E-02	↓

Feature name	Nano-ESI-MS raw p value	Nano-ESI-MS direction	LC-MS raw p value	LC-MS direction
Methyl propenyl ketone,HMDB0001184			3.16E-03	↑
Methylgingerol,HMDB0029852			3.92E-02	↑
Monoisobutyl phthalic acid,HMDB0002056			1.59E-02	↑
N-(2,4-Eicosadienoyl)piperidine,HMDB0032001			1.76E-04	↑
N-Acetyl-2,3-dihydro-1H-pyrrole,HMDB0031163	8.65E-02	↑		
N-Acetylhistamine,HMDB0013253	3.14E-02	↑	4.17E-03	↓
N-Desmethyl tapentadol,HMDB0060611	5.84E-03	↑		
N-Methylphenylethanolamine,HMDB0001387	3.77E-02	↑		
N-Succinyl-L,L-2,6-diaminopimelate,HMDB0012267			5.38E-02	↓
N-Undecanoylglycine,HMDB0013286	8.18E-03	↑	1.22E-02	↓
N5-Hexanoylspermidine,HMDB0029568			5.08E-02	↑
Neuroprotectin D1,HMDB0003689			4.19E-02	↓
Nookatone,HMDB0013687			7.57E-02	↑
Nootkatol,HMDB0013688			6.67E-03	↑
Octanal,HMDB0001140			9.49E-02	↑
Palmitaldehyde,HMDB0001551			4.80E-02	↑
Panthenol,HMDB0004231	6.53E-02	↑		
Pentadecanoic acid,HMDB0000826			9.36E-02	↑
Polypropylene glycol (m w 1,200-3,000),HMDB0032478	8.58E-03	↑	9.49E-03	↓
Proline,HMDB0000162	4.49E-02	↑		
Propofol,HMDB0014956			6.42E-03	↑
Pseudooxynicotine,HMDB0001240			4.34E-02	↑
Pyroglutamic acid,HMDB0000267	5.66E-02	↑		
SM(d16:1/18:2(10E,12Z)+=O(9)),HMDB0290277			5.73E-02	↓
Saccharopine,HMDB0000279	5.52E-02	↑		
Serylvaline,HMDB0029052			9.02E-02	↑
Terbutaline,HMDB0015009	1.31E-02	↑		
Tetrahydrofuran,HMDB0000246			6.34E-03	↑

Feature name	Nano-ESI-MS raw p value	Nano-ESI-MS direction	LC-MS raw p value	LC-MS direction
Tiglylcarnitine,HMDB0002366	4.02E-03	↑		
Tridecanal,HMDB0030928			8.35E-02	↑
Trigoforin,HMDB0029495			3.60E-02	↑
Tripropylamine,HMDB0032545			2.84E-03	↓
Tryptamine,HMDB0000303	2.77E-02	↑		
Tyramine,HMDB0000306	7.20E-02	↑		
Uracil,HMDB0000300	8.77E-02	↑		
alpha-CEHC,HMDB0001518			9.71E-03	↑
beta-Damascenone,HMDB0013804			2.33E-02	↑
chondroitin sulfate E (GalNAc4,6diS-GlcA), precursor 5a,HMDB0062464	6.13E-02	↑		
gamma-Glutamylglutamic acid,HMDB0011737			4.26E-02	↓
p-Cresol,HMDB0001858	2.41E-02	↑		
xi-p-Menth-3-ene,HMDB0037213	2.22E-02	↑		
